# High Frequency Oscillations in Rat Hippocampal Slices: Origin, Frequency Characteristics, and Spread

**DOI:** 10.3389/fneur.2020.00326

**Published:** 2020-04-22

**Authors:** Isaac Naggar, Mark Stewart, Rena Orman

**Affiliations:** ^1^EEG Section, NINDS, National Institutes of Health, Bethesda, MD, United States; ^2^Department of Neurology, State University of New York Downstate Medical Center, Brooklyn, NY, United States; ^3^Department of Physiology and Pharmacology, State University of New York Downstate Medical Center, Brooklyn, NY, United States

**Keywords:** CA3c, ripples, very fast oscillations, bicuculline, kainic acid, carbenoxolone, gap junctions, electrode array

## Abstract

Field potential oscillations reflect repetitive firing and synaptic activity by ensembles of neurons in certain areas of the brain. They can be distinguished as slow (e.g., alpha, delta, and theta), fast (e.g., beta and gamma), and high frequency oscillations (HFOs). Neuronal oscillations are involved in a variety of physiological and pathophysiological states such as cognition, consciousness, and seizures. The laminar structure of rat hippocampus affords a way to study these oscillations in hippocampal slices. Rat ventral hippocampal brain slices were cut and maintained in a recording chamber that permitted 64 simultaneous extracellular recordings in the presence of artificial CSF. Brief single stimulus pulses were applied with bipolar electrodes to the CA3 or CA1 regions of hippocampus. Single pulses triggered epileptiform population events that included HFOs in the 150–250 Hz range in the presence of GABA_A_ receptor blockade or kainic acid. HFOs also occurred spontaneously in the presence of kainic acid. The oscillations had the largest amplitude in the CA3c cell layer, regardless of the drug, and were synchronized throughout the cell layer. AMPA receptor blockade stopped these HFOs, whereas NMDA receptor blockade did not. Gap junction activation did not restore HFOs in the presence of AMPA receptor blockade. Our findings suggest that a population of excitatory neurons in CA3c may be a primary focus of seizure-like activity in Ammon's Horn. We suggest that the interconnection of CA3c is different from the rest of CA3.

## Introduction

Field potential oscillations reflect synchronized rhythmic synaptic potentials and/or firing by populations of neurons. High frequency oscillations (HFOs), in some studies referred to as “ripples,” exist in the 80–600 Hz range. It has been proposed that this broad frequency range reflects different kinds of activity, and recent reviews have outlined the possibilities for HFO generation involving synaptic and non-synaptic mechanisms as well as the challenges associated with identification of mechanism in brain (1, 2). HFOs can be observed in limbic structures and all over neocortex (3–6) in both pathologic contexts like seizures (7, 8) and in normal contexts such as cognition and sleep (9, 10). The oscillatory periods tend to be of shorter duration and amplitude on account of the neuronal synchrony necessary to achieve them (11).

Pathologic HFOs tend to be of higher frequency than physiologic HFOs (7) and are thought to be a feature of the seizure onset zone in patients with epilepsy (12, 13). HFOs of around 200 Hz have been described under normal conditions in the CA1 pyramidal cell layer of awake immobile rats.

Population bursts of the CA3 network occurring during eating, drinking, slow-wave sleep, and awake immobility are thought to be field excitatory post-synaptic potentials (EPSPs) that depolarize CA1 pyramidal cells via the Schaffer collaterals (10) and the dentate gyrus (14). These in turn are thought to produce the HFOs in the 200 Hz range in normal rats (9). The bursts of sinusoidal activity last 5 to 15 cycles with peak-to-peak amplitudes less than 500 microvolts (10).

Laminar profiles of these oscillations have shown that the oscillations restrict themselves to the pyramidal cell layer with almost no phase lag over 2 mm distance (15), even up to 5 mm in the rat (10). The ability to extend over this amount of space essentially shows there is an underlying network that must generate the oscillations, as it cannot arise from single neurons with propagation from cell to cell (16).

Two main hypotheses have been offered as the mechanism for these oscillations (1, 2). One is that there is a synaptic basis for the oscillations with both excitatory and inhibitory control (9, 15). The other hypothesis states that gap junctions are responsible for the oscillations (17, 18). A third and contributing theory posits there may be some role for local field effects in the amplitude of the oscillation (19).

Evidence supporting a synaptic mechanism has shown that the oscillations are related to variations in pyramidal cell and interneuron activity (20). High frequency 200 Hz oscillations within CA1 reflect synchronized IPSPs in the perisomatic region of CA1 pyramidal cells (15). The probability of pyramidal cell firing is greatest during the negative peaks of the oscillations, indicating a degree of excitatory synchrony. Thus, long-range inhibitory control superimposed over a depolarizing input can produce synchronized oscillations (10). In addition to high-density connection basket cells that produce local inhibition, long-range inhibitory control via interneurons with axonal length of 20 to 100 mm has been described (21). Evidence against the synaptic hypothesis includes the presence of 150–200 Hz oscillations in the absence of extracellular calcium ions, which are required for chemical synapses (17).

Evidence for the gap junction hypothesis includes abolition of the HFOs in the presence of gap junction blockers, including halothane, carbenoxolone, and octanol (17). However, multiple blockers have been used since specific gap junction blockade has not been achieved (22). Spontaneous HFOs have been shown to be less frequent in connexin 36-deficient mice (22). There is also evidence of electrical coupling between hippocampal principal cells (23–25), which suggests the presence of gap junctions. The oscillations are thought to arise via gap junctions between axons of pyramidal cells (18). In one study, gap junctions were identified in mossy fibers in CA3b (total of 10 axoaxonic pairs) and CA3c (one axoaxonic pair) using electron microscopy and immunogold labeling (26).

In a study of mouse brain slices, D'Antuono et al. (27) showed that HFOs occurred in slices disinhibited with picrotoxin, depended on non-NMDA glutamatergic receptors, did not depend on gap junction availability, and could occur in isolated dentate gyrus sub-slices. These authors did note that initiation of HFO/ripple activity appeared to be in either CA3 or entorhinal cortex, depending upon the particular slice being studied. These results point away from inhibitory circuits or gap junctions for HFO generation.

We sought to explore the origins of HFO in rat brain slices where would could apply 64-electrode array recordings to define the spatio-temporal distribution of high frequency oscillations and relate them to their inhibitory and excitatory controls.

## Materials and Methods

All procedures were approved by the University's Animal Care and Use Committee and conform to NIH guidelines.

### Slice Preparation and Maintenance

Male Sprague-Dawley albino rats (150–200 g; 3–5 weeks old) were anesthetized with halothane and decapitated. Each brain was removed from the skull, bisected, and placed briefly in ice cold artificial CSF. Thick slices of tissue (about 1–2 mm thick) were cut horizontally from the intact hemispheres with its dorsal face at about the level of the hippocampal genu. These thick sections were mounted in a Leica VT1000S sectioning system (Leica; Nussloch, Germany), which was used to cut brain slices for physiological study (350–400 μm). Final slices were simple horizontal sections trimmed with a cut perpendicular to the midline on the rostral side of area CA3 and the level of the slices corresponded to a range of about 2.6–4.6 mm above the interaural line (28). Slices were maintained in a holding chamber containing oxygenated artificial cerebrospinal fluid (see below).

From the holding chamber, single slices were placed in the MED64 chamber (Panasonic MED64; Osaka, Japan). The MED64 chamber is a 22 mm diameter well formed from a plastic ring cemented to a glass base that contains the electrodes. Conductive strips embedded in the glass base terminate in platinum-platinum black electrodes that are nearly flush with the well floor. Flow is regulated such that slices are just below an interface configuration. The perfusion solution (1 ml/min) was composed of (in mM): NaCl 125, KCl 2.5 to 5, CaCl_2_ 1.7, MgCl_2_ 1.2, NaHCO_3_ 26, and glucose 10; pH 7.4 when exposed to 95% O_2_ and 5% CO_2_. The temperature of the MED64 chamber was maintained at 30°C by warming the perfusate with an inline heater. The ventral horizontal slice preparation contains area CA1 and many of the surrounding areas, including: CA3, subiculum, presubiculum, and entorhinal cortex (29–31).

### Recording and Stimulating Techniques

The MED64 chamber allows simultaneous extracellular recordings from 64 electrodes (50 μm squares). Each electrode is a platinum black-plated square embedded in the floor of the recording chamber. Inter-electrode distances (center to center) were 100, 150, or 300 μm. Recording electrode impedances are 22 kΩ (at 1 kHz) and each is referred to a set of four reference electrodes in the periphery of the chamber that are electrically connected. The recording electrodes are arranged in an 8 x 8 array embedded on the bottom of the chamber. Brief stimulating pulses were delivered using platinum-iridium parallel bipolar stimulating electrodes (150 μm tip separation; FHC; Bowdoinham, ME) with <100 kΩ electrode impedances. Stimuli were biphasic pulses (50–100 μs in total duration) applied to the CA3 or CA1 regions of hippocampus through constant current stimulus isolation units. The bipolar stimulating electrode was placed from the top side of the slice. Data were digitized at 20 kHz per channel and stored on disk using MED64 Conductor software. Events could be viewed offline using the MED64 Conductor software.

### Pharmacology

All drugs were applied to the bath by adding them to the perfusate reservoir. The concentrations given are concentrations that exist in the reservoir and were achieved in the recording chamber over a period of minutes. Recordings in the presence of all drugs were taken after sufficient time for equilibration in the recording chamber. Equilibration was apparent in recordings as a change in evoked response. Bicuculline (bicuculline methiodide; 50 μM), AP-5 (DL-(2)-2- amino-5-phosphonopentanoic acid; 40 μM), CPP (3 ((RS)-2-carboxypiperazin-4-yl)-propyl-1-phosphonic acid; 20 μM), carbenoxolone (100 μM) and CNQX (6-cyano-7-nitroquinoxaline-2,3-dione or 6-cyano-7-nitroquinoxaline-2,3-dione disodium; 20 μM), and trimethylamine (TMA; 4 mM) were obtained from Sigma (Sigma-Aldrich; St. Louis, MO). Kainic acid (15 nM) was obtained from Abcam (Cambridge, MA). Some batches of CNQX were obtained from Tocris Bioscience (Ellisville, MO). Bicuculline was used to antagonize GABA_A_ receptors, and kainic acid was used as a kainate receptor agonist. AP-5 and CPP were used as NMDA receptor antagonists, and CNQX was used as an AMPA receptor antagonist. Carbenoxolone was used to block gap junctions and TMA was used to activate gap junctions.

### Data Analysis

Analysis of the electrode recordings was done using Matlab with the Signal Processing Toolbox (Mathworks; Natick, MA) as well as with the Joint Time-Frequency Analyzer software (National Instruments; Austin, TX).

Color spectrograms of raw data from individual recordings were made using the Joint Time Frequency Analyzer software. The recordings chosen were the ones with greatest amplitude oscillation, as found by using the Fast Fourier Transform (FFT). High frequency oscillations were noted to all be within the 150–250 Hz range, and beginning and end times of the oscillations were found using a set threshold amplitude within this range from instantaneous FFTs. The time of maximum oscillatory amplitude in the 150–250 Hz range was also located. In total, 20 slices (17 rats) with oscillations after application of bicuculline and 13 slices (10 rats) after application of kainic acid (all slices under electrical stimulation) were studied in this way. A total of 6 slices (4 rats) with kainic acid displayed spontaneous oscillations, and these were also analyzed in the same fashion.

The rest of the data analysis was conducted with Matlab. Descriptive statistics of each slice among all electrodes included (1) the time of greatest oscillatory activity, (2) the amplitude of the oscillation given by the power of the FFT, and (3) the frequency of the oscillatory activity. As a measure of sustainability of the oscillation, (4) the amount of time from greatest oscillatory activity to the end of the oscillation was calculated. For a given slice, the above parameters were calculated for each of the 64 channels over 8 representative sweeps, which could be found in a subset of the slices (An exception was made in 2 of the slices with spontaneous oscillations in kainic acid, for which only 5 or 6 events could be recorded). The results were subsequently averaged across the 8 sweeps for each channel. A series of short time Fourier transforms (STFTs) were calculated for each channel to identify the time of peak oscillatory activity in the high frequency oscillation frequency range. The presence of high frequency oscillations was determined with an amplitude threshold of the FFT in the HFO range that was initially verified manually as the absence of significant oscillation. The 13 ms of data before and after the calculated time were mean detrended, and the point at which the oscillations reached an absolute maximum in magnitude was taken to be the precise peak time of oscillations. The amplitude of the oscillations was determined as the amplitude of the FFT at that time. Alternatively, the voltage difference between the largest peak and valley of the oscillations was used as a measure of the amplitude of the oscillations; this was done to compare slices with bicuculline or kainic acid that had CPP or AP-5 added to them. To calculate the frequency, the three oscillations before and after the peak oscillatory time were located using a threshold-lockout algorithm and their frequency was averaged. The end oscillatory time was found by taking consecutive FFTs after the peak oscillatory time and finding the first FFT over the 150–250 Hz range to return as below the set threshold.

The above methods could not be used for the slices to which kainic acid was applied, since some of them had their peak time too early in the sweep. Instead, the methods described below were used. Results from these calculations were validated by applying them to data from bicuculline-treated slices and comparing to existing calculations from the previous methods described. As with the slices in the bicuculline bath, calculations were averaged over 8 sweeps of the 64-channel data. Each sweep and channel had to have the beginning and end of oscillatory activity identified by hand after applying a band-stop filter of 0–70 Hz. The sum of the absolute value of the points between the selected points was taken as the full-wave rectified area under the curve. This value was divided by the length of time of oscillation to yield average amplitude, or intensity, of the oscillations. The peak time of oscillation was taken as the point at which the oscillation reached its maximum absolute value. The length of the oscillations from peak to end of the oscillations could then also be determined. The frequency of the oscillations was determined by taking the frequency with maximum amplitude in the FFT of the entire oscillatory period.

Color maps of oscillation intensity over all 64 electrodes were also made. These could be superimposed upon the slice images to appreciate the areas of greatest oscillatory activity. Depth profile plots of a single sweep were made by examining an electrode row or column of interest perpendicular to the cell layer. The signals from these channels were mean detrended, band-stop filtered (0–70 Hz), and subsequently plotted. The voltage at a specific point in time was taken as the value of the filtered data at that time point for each of the electrodes. In a manner similar to the depth profiles, profile plots along the cell layer were made. Electrodes along the cell layer were located, and their data was band-pass filtered (70–350 Hz) and subsequently plotted. Peaks and valleys were found within the oscillations of each electrode using a threshold-lockout algorithm.

Cross-correlation between electrodes along the cell layer and the electrode with maximal oscillatory activity was calculated. First, for each sweep analyzed, the collection of electrodes along the cell layer was identified. Four sweeps from each slice were taken and band-stop filtered (0–70 Hz). Using MATLAB's xcorr function, the *r*^2^ and lag values were calculated for the electrodes compared to the electrode with most oscillatory activity. For bicuculline-treated slices, the data used was from the peak of the oscillation until its end of each individual channel. Data from the beginning to end of the oscillations in kainic acid-treated slices could be used. Maximum *r*^2^ values with their associated lag times were taken. The resulting values were averaged across the sweeps. Distances between each of the electrodes in the slices were calculated, which allowed for creating a composite correlation using data from all slices.

Data are reported as means ± SD, unless the measurements are means themselves, in which case data are reported as means ± SEM. All statistics were computed with Minitab 18 (Minitab, Inc., State College, PA, USA). Unless otherwise noted, parameters calculated from slices with bicuculline or kainic acid (triggered or spontaneous) were compared using ANOVA with Tukey's *post-hoc* analysis. Paired *t*-tests were used to compare bicuculline- and kainic acid-treated slices before and after the addition of CPP or AP-5. In general, a *p*-value < 0.05 was considered to be statistically significant. Significant *p*-values are denoted in figures with asterisks (^*^).

## Results

### Spatio-Temporal Description of Hippocampal HFOs

**Figure 1:**
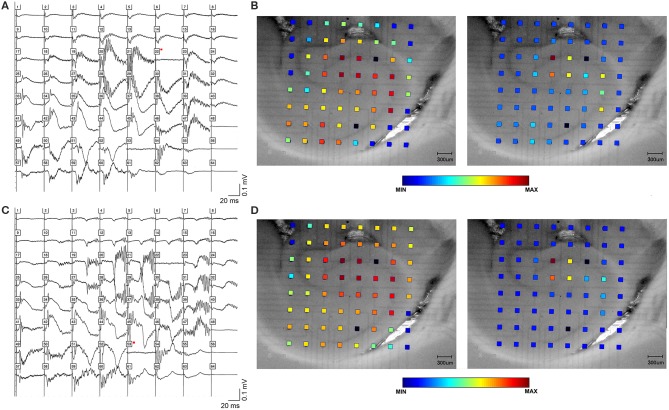
High frequency oscillations in the presence of bicuculline. HFOs can be observed in rat hippocampal slices containing CA3, CA1, subiculum and dentate gyrus. **(A)** depicts field potentials evoked by a single stimulus pulse applied in area CA3 (black square in the cell layer). **(B)** Maximum amplitude and frequency were recorded in area CA3. The color grid indicates the exact location of recording electrodes. The dark red color of the calibration spectrum represents the maximum oscillation amplitude (right panel) and maximum log of the amplitude (left panel) for the CA3 stimulating site. **(C,D)** show the same oscillations, this time evoked by a single pulse applied at stimulating site in area CA1 (black square in the proximal stratum radiatum of mid-CA1, i.e., close to the cell layer).

**Figure 2:**
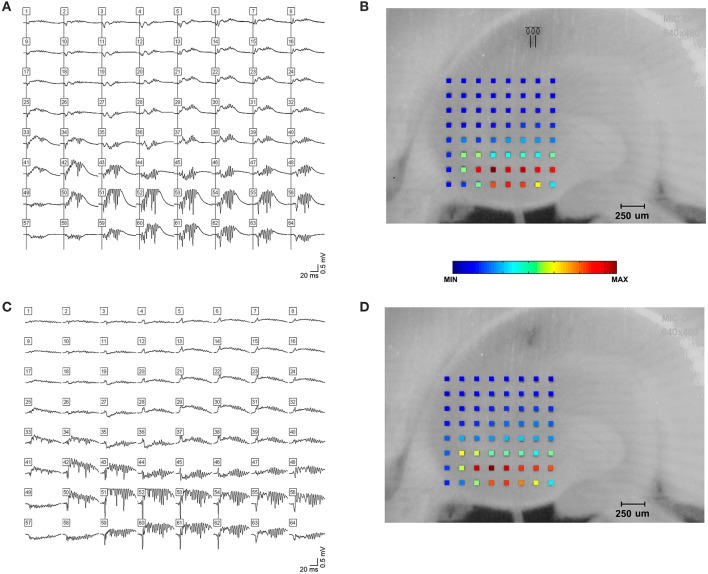
High frequency oscillations in the presence of kainic acid. HFOs could be observed in rat hippocampal slices bathed in kainic acid. **(A)** shows field potentials evoked by a single stimulus pulse, which is shown in **(B)**. Stimulus electrode location is distal stratum radiatum in mid-CA1, i.e., away from the cell layer. Raw amplitude data is denoted by the color spectrum of the electrode grid. **(C,D)** demonstrate spontaneous high frequency oscillations in the presence of kainic acid.

**Figure 3:**
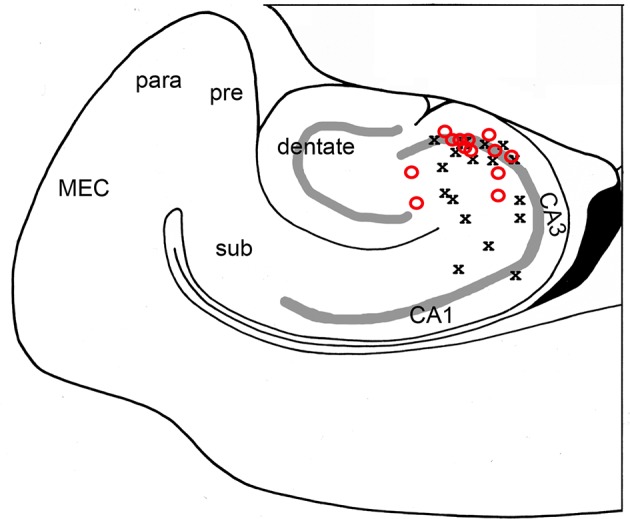
High frequency oscillations are maximal in area CA3c. The channel with greatest oscillatory activity was found for each slice and correlated with its anatomical position. Both bicuculline- (red O; *N* = 17 slices) and kainic acid- (black X; *N* = 13 slices) treated slices showed maximal oscillatory in area CA3c, most notably in the cell layer.

A total of 41 animals were used, with 41 bicuculline-treated slices (31 rats) and 13 kainic acid-treated slices (10 rats). Single pulses in the presence of bicuculline (Figure 1) or kainic acid (Figure 2) triggered epileptiform events that contained episodes of high frequency oscillations lasting 50–150 ms. Of the kainic acid-treated slices, 6 slices (4 rats) had spontaneous oscillations for at least 5 sweeps (Figure 2). Stimulation at CA3 and CA1 produced similar responses (Figure 1), with oscillations of maximal amplitude in area CA3c for both kainic acid—and bicuculline—treated slices (Figure 3).

**Figure 4:**
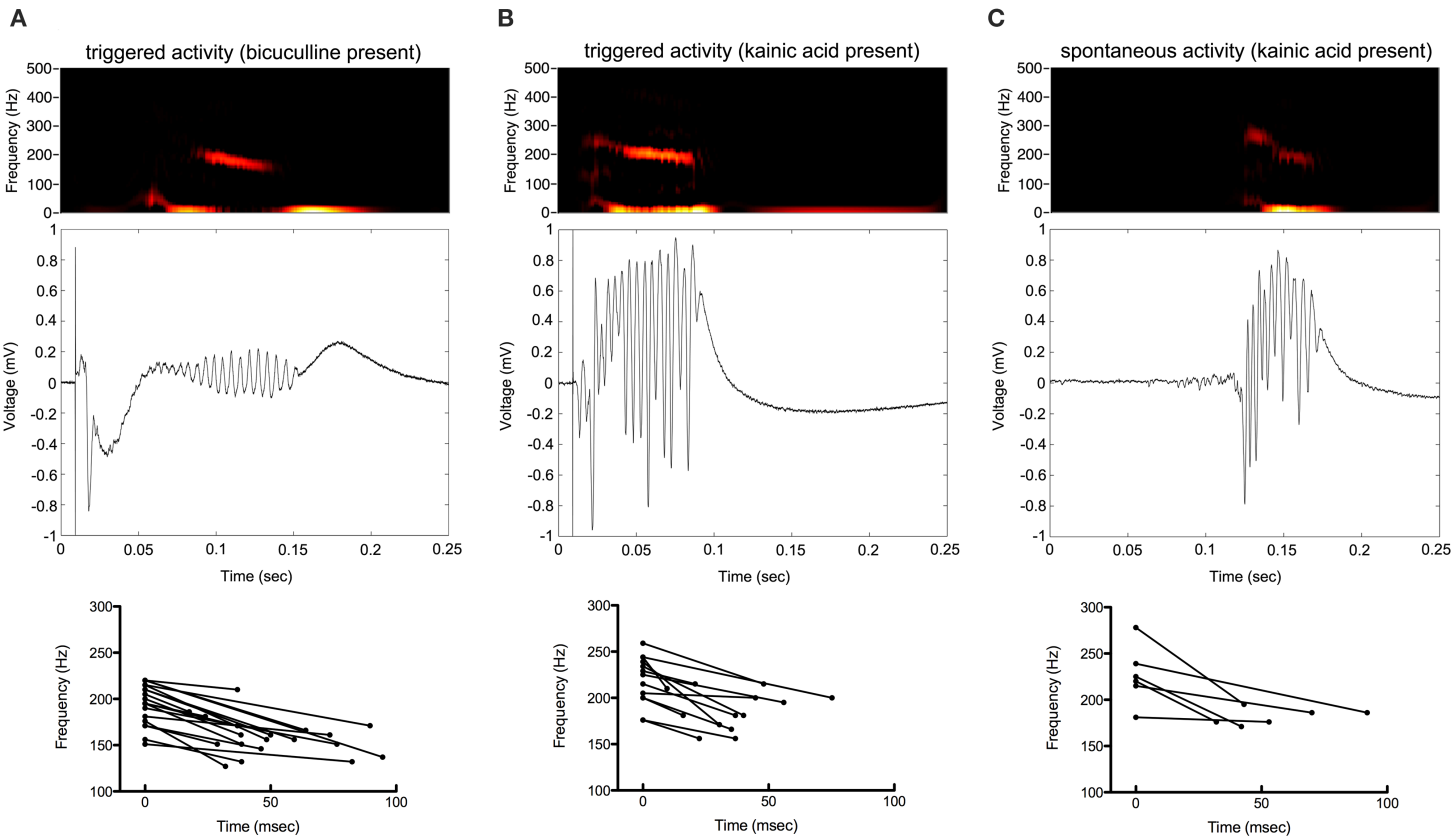
Frequency of the oscillations decrease over time. Color spectrograms and raw data of representative recordings from the electrode with greatest oscillatory activity are shown in **(A–C)**. Start and end frequencies, as well as duration of oscillatory activity over time, are shown determined from the electrodes with greatest oscillatory activity from each of the slices.

Oscillations began 25–125 ms from the beginning of stimulation. They appeared to be maximal in frequency at their beginning and decrease in frequency over time. The amplitude of oscillations was maximal in the middle of the oscillatory period (Figure 4).

**Figure 5:**
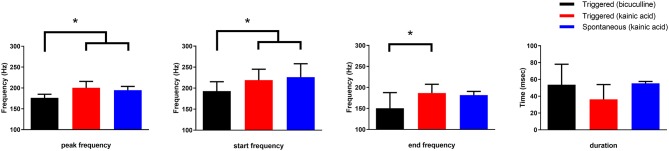
Frequency and duration characteristics of oscillations in the presence of bicuculline or kainic acid. Slices with kainic acid (either triggered events or spontaneous) had a higher starting and peak frequency than those slices with triggered events in bicuculline after *post-hoc* analysis (*p < 0.004 and 0.001, respectively). The frequency at the end of the oscillations were different for only the triggered bicuculline and kainic acid slices after *post-hoc* analysis (*p = 0.004). There was no difference in duration of the oscillations, although there was a trend toward a difference between spontaneous and triggered oscillations in the presence of kainic acid (*post-hoc* analysis *p* = 0.058).

The frequency of HFOs and the duration of the oscillatory period were measured at the channel in which the oscillations had greatest amplitude (Figure 5). Peak frequency was 196 ± 22, 227 ± 21, and 233 ± 24 Hz for bicuculline triggered (*n* = 20; 17 rats), kainic acid triggered (*n* = 13; 10 rats), and kainic acid spontaneous events (*n* = 6; 4 rats), respectively. There was no difference in duration of the oscillations (bicuculline triggered 54 ± 24 ms vs. kainic acid triggered 36 ± 18 ms vs. kainic acid spontaneous 55 ± 22 ms), although there was a trend toward a difference in these groups *p* = 0.07. *Post-hoc* analysis revealed the trend to be a difference in the triggered bicuculline and kainic acid treated slices (*p* = 0.08).

**Figure 6:**
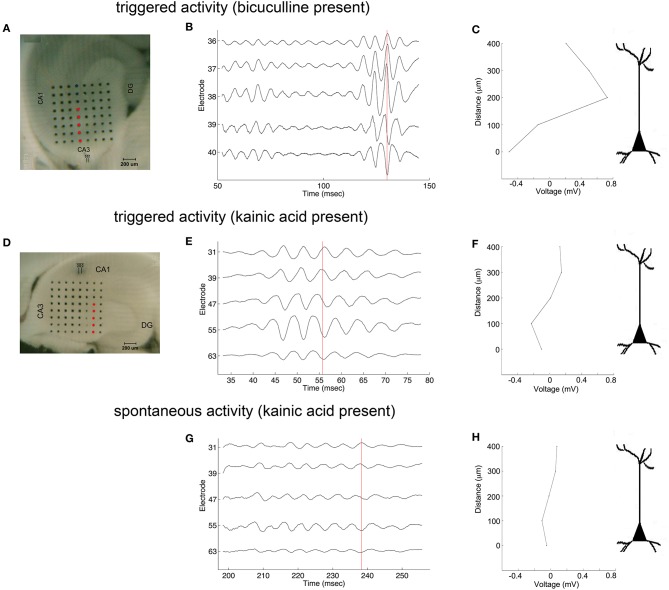
Coarse voltage depth profile analysis in the presence of bicuculline or kainic acid. **(A,D)** show images of slices in bicuculline and kainic acid, respectively. Recordings of electrodes marked in red in those images are shown in **(B,E,G)** for triggered activity in bicuculline, triggered activity in kainic acid, and spontaneous activity in kainic acid, respectively. **(C,F,H)** plot the raw voltages marked by the red lines going through the records in **(B,E,G)**, respectively to show a phase reversal over the cell layer. Stimulus location in **(A)** is the white matter near the fimbria, below the cell layer. Stimulus location in **(D)** is stratum radiatum in mid-CA1.

**Figure 7:**
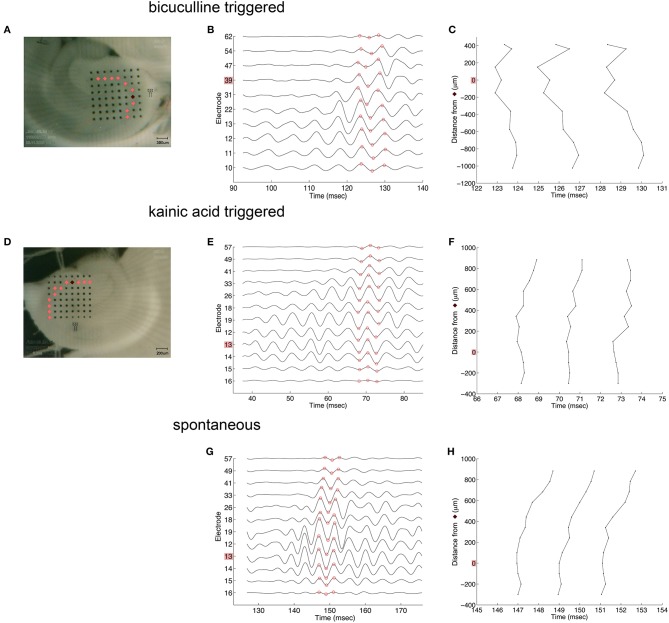
Coarse voltage profile analysis over the cell layer in the presence of bicuculline or kainic acid. **(A,D)** show images of slices in bicuculline and kainic acid, respectively. Recordings of electrodes over the cell layer marked in pink in those images are shown in **(B,E,G)** for triggered activity in bicuculline, triggered activity in kainic acid, and spontaneous activity in kainic acid, respectively. The electrodes marked in dark red in **(A,D)** denote the sites of greatest overall oscillatory activity. **(C,F,H)** plot the time at which the recordings reach their peaks and troughs [marked in red circles in **(B,E,G)**, respectively]. These show little to no lag of the oscillation along the cell layer. Stimulus location in **(A)** is the white matter near the fimbria, below the cell layer. Stimulus location in **(D)** is stratum radiatum in mid-CA1.

Oscillations appeared to originate from the cell layer in both bicuculline- and kainic acid-treated slices (Figure 6). Depth profile analysis showed reversal of the voltage just apical to the cell layer of CA3c or other segments of CA3. The apical negativity in voltage profiles is consistent with excitatory synaptic activity and was consistent across spontaneous and evoked HFOs seen in disinhibited slices or slices activated by kainic acid. Moving along the cell layer, the temporal shifting of peaks suggests a spread velocity of ≤ 1 m/s (1 mm/ms) (Figure 7), also consistent with spread times for epileptiform activity in hippocampus [e.g., (32)].

**Figure 8:**
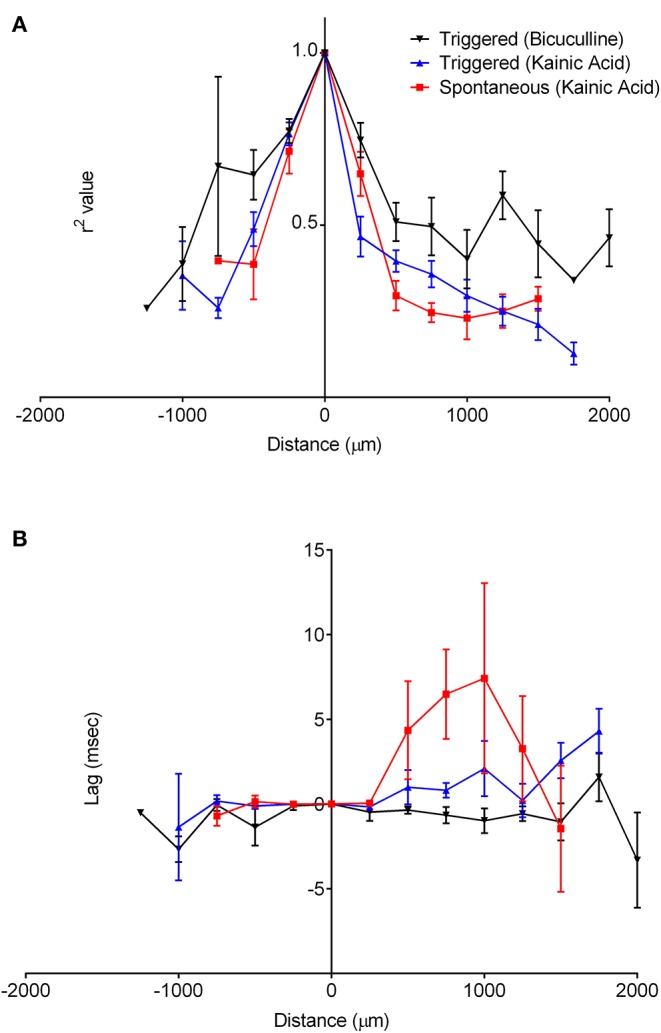
Correlation of oscillations with the origin site in CA3. The recordings along the cell layer were compared with recordings from the maximal oscillatory activity for all slices. **(A)** shows that for recordings with both drugs, correlation of the oscillations decreases as a function of distance over the cell layer. It appears to decrease faster in slices bathed in kainic acid than those in bicuculline. The lag time compared to the maximal oscillatory electrode appeared to be constant over the cell layer, as in **(B)**.

The oscillatory coherence decreased with distance from the channel of maximal oscillatory amplitude along the cell layer (Figure 8A), yet with little to no lag in all slices except for kainic acid-treated slices with spontaneous oscillation (Figure 8B). There appeared to be greater correlation over larger distances among bicuculline-treated slices than kainic acid-treated slices.

**Figure 9:**
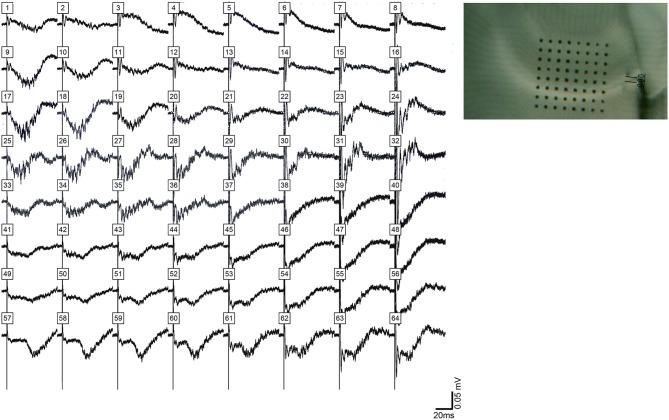
HFOs evoked in CA1 after removal of CA3 from the slice. **(Left)** shows filed potentials evoked by a single stimulus pulse applied to the alveus on the CA3 (proximal) side of CA1 after area CA3 was separated from the slice by knife cut, which is shown in **(Right)**. The amplitude and synchrony of HFOs in CA1 under these conditions was less than HFOs observed in CA3 or in CA1 in intact slices.

We made recordings of evoked HFOs in CA1 of 13 slices (6 rats) after CA3 was cut off from the rest of the slice. Oscillations occurred as part of triggered events in CA1, but these were not as well synchronized as events recorded in CA3 or in CA1 of intact slices. This is illustrated in Figure 9.

### Pharmacology of Hippocampal HFOs

Comparison of all channels in slices in bicuculline and kainic acid was undertaken. The time to peak oscillatory activity after electrical stimulation was greater in the bicuculline-treated slices than in the kainic acid-slices (Table 1; 80 ± 9 vs. 33 ± 6 ms; Student's *t*-test, *p* = 0.003). HFOs in bicuculline were slower than those in kainic acid in general (*p* < 0.002). The time from the peak frequency to the end of the oscillation was not different between groups, but spontaneous oscillations appeared to last longer than triggered ones in kainic acid (*p* = 0.058).

**Table 1:** Characteristics of hippocampal triggered and spontaneous VFOs in presence of bicuculline or kainic acid.

	**Triggered Bic (*N* = 12)**	**Triggered KA (*N* = 11)**	**Spontaneous KA (*N* = 6)**	***p*-value (B-KA)**	***p*-value (B-KAs)**	***p*-value (KA-KAs)**	***p*-value (all)**
Peak time of oscillatory activity (ms)	80 ± 9	33 ± 6	N/A	N/A	N/A	N/A	0.0003
Frequency of peak oscillatory activity (Hz)	172 ± 2	182 ± 2	184 ± 2	0.001	0.002	0.828	0.0003
Time to end of oscillation (ms)	42 ± 3	33 ± 4	50 ± 8	0.31	0.47	0.058	0.065

*Bic, bicuculline; KA, kainic acid (triggered); KAs, kainic acid (spontaneous)*.

Comparison of oscillations before and after application of CPP to 6 slices (6 rats) bathed in bicuculline was performed. Also, in slices bathed in bicuculline (6 slices; 5 rats) and kainic acid (5 slices; 5 rats), the effect of application of AP-5 was evaluated. As shown in Table 2, there was significant increase in frequency after application of CPP to slices bathed in bicuculline (174 ± 3 vs. 184 ± 3 Hz; *p* = 0.0033). Increase in frequency was also observed after application of AP-5 to slices bathed in bicuculline (169 ± 3 vs. 178 ± 3 Hz; *p* = 0.011), and the time to the end of the oscillation was decreased (46 ± 3 vs. 36 ± 3 ms; *p* = 0.043). There was no change in any of measured parameters to slices bathed in kainic acid after application of AP-5.

**Table 2:** Effect of CPP or AP-5 to slices bathed in bicuculline or kainic acid.

	**Bicuculline**	**CPP**	***p*-value**
**Bicuculline + CPP (*N* = 6)**			
Peak time of oscillatory activity (ms)	73 ± 13	61 ± 7	0.32
Amplitude of oscillatory activity (au)	13 ± 9	20 ± 14	0.30
Peak-to-valley oscillation amplitude (mV)	0.19 ± 0.04	0.22 ± 0.05	0.16
Frequency of peak oscillatory activity (Hz)	174 ± 3	184 ± 3	0.0033
Time to end of oscillation (ms)	37 ± 5	28 ± 2	0.17
	**Bicuculline**	**AP-5**	***p*****-value**
**Bicuculline + AP-5 (*N* = 6)**			
Peak time of oscillatory activity (ms)	86 ± 12	92 ± 8	0.50
Amplitude of oscillatory activity (au)	10 ± 3	15 ± 4	0.14
Peak-to-valley oscillation amplitude (mV)	0.18 ± 0.02	0.20 ± 0.02	0.24
Frequency of peak oscillatory activity (Hz)	169 ± 3	178 ± 3	0.011
Time to end of oscillation (ms)	46 ± 3	36 ± 3	0.043
	**Kainic acid**	**AP-5**	***p*****-value**
**Kainic acid + AP-5 (*N* = 5)**			
Peak time of oscillatory activity (ms)	26 ± 4	26 ± 4	0.87
Amplitude of oscillatory activity (au)	486 ± 111	597 ± 213	0.54
Peak-to-valley oscillation amplitude (mV)	0.37 ± 0.06	0.58 ± 0.13	0.11
Frequency of peak oscillatory activity (Hz)	192 ± 13	192 ± 12	0.93
Time to end of oscillation (ms)	44 ± 10	28 ± 3	0.22

**Figure 10:**
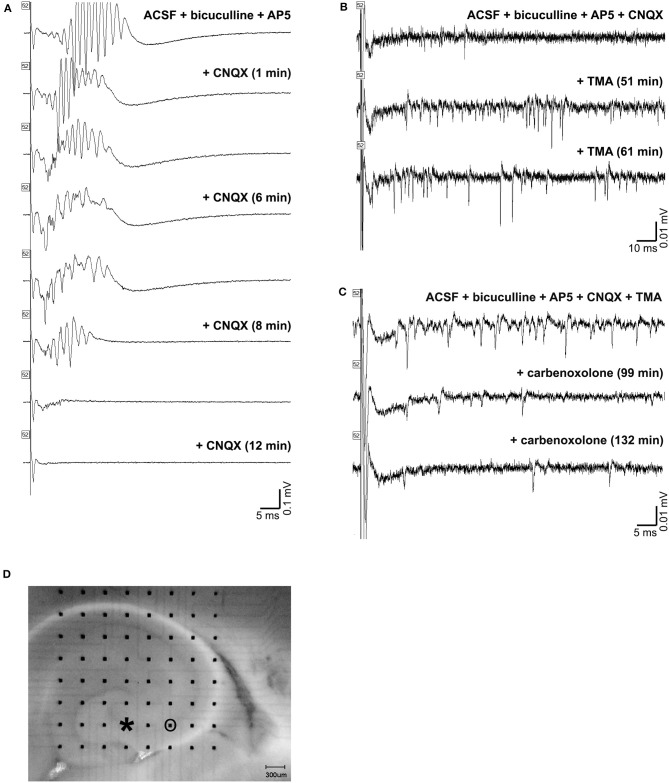
Gap junction and synaptic control over high frequency oscillations. **(A)** shows a triggered HFO in the presence of bicuculline and AP-5 in the top tracing. Addition of CNQX caused cessation of the oscillations in the bottom tracings. The addition of TMA to these slices increased activity, but did not restore high frequency oscillations, as shown in the tracings in **(B)**. Further addition of carbenoxolone abolished the increased activity, as in the tracings in **(C)**. An image of the slice used is shown in **(D)**. The electrode used for stimulation is marked with an asterisk (at the end of CA3 inside the hilus), and the electrode used for recordings in **(A,B,C)** is circled.

Application of CNQX to bicuculline-treated slices caused cessation of the oscillations (Figure 10A). A desynchronization effect similar to that described by Foffani et al. (33) is evident as the CNQX effect develops. Addition of TMA to these slices increased spontaneous single and multi-unit spiking activity, but did not restore high frequency oscillations (Figure 10B). And, further addition of carbenoxolone abolished the single and multi-unit spiking activity (Figure 10C). The addition of carbenoxolone to slices with only bicuculline did not cause cessation of the oscillations, but it did decrease the frequency of spontaneous episodes of oscillatory activity during recordings. In 8 slices (8 rats), carbenoxelone tested in bicuculline-exposed slices did not disrupt HFOs. Interestingly, the only effect that we detected was that occasionally, the stimulus trigger pulse did not trigger a population event. The maximal failure rate was 1 failure/3 stimulus trigger pulses. Population events that did occur were indistinguishable in duration, amplitude, or frequency characteristics from events triggered in the presence of bicuculline only.

## Discussion

Using the laminar characteristic of the rat hippocampus, the rat hippocampal slice model is ideal for studying HFOs in hippocampus. We found HFOs to occur either after direct electrical stimulation in the presence of GABA_A_ receptor blockade or kainic acid, or spontaneously in the presence of kainic acid. These oscillations had the largest amplitude and earliest onset in area CA3c cell layer, regardless of the drug, and their synchronization/spread extended over distances greater than 1 mm. The frequency of the oscillations was in the 150–250 Hz range, and the frequency decreased over time within a single oscillatory epoch. HFOs also tended to be higher frequency and the oscillatory period lasted longer in the presence of kainic acid than in the presence of a GABA_A_ receptor blocker. NMDA antagonism did not significantly alter oscillations either in the presence of GABA_A_ blockade or in kainic acid, except for a small increase in the frequency of the oscillations. Oscillations appeared to require AMPA receptor activity, as the HFOs stopped in bicuculline with the addition of an AMPA receptor antagonist, although there was still action potential activity in the slices. Addition of a gap junction opener increased the single and multi-unit action potential activity, but did not restore HFOs. Collectively, we conclude that ≈ 200 Hz HFOs depend upon glutamatergic synaptic transmission for synchronization of action potentials generated by various mechanisms, including disinhibition, convulsant action, and possibly the presence of gap junctions. Disruption of either the mechanism of synchronization or the action potential activity substrate to be synchronized can eliminate these HFOs.

### Importance of CA3c in High Frequency Oscillations

Others have recorded HFOs simultaneously in areas such as CA3 and CA1 (34) in normal behaving rats or in areas such as entorhinal cortex, dentate gyrus, and CA3 in disinhibied brain slices (27), this is the first study of HFOs in rat hippocampus with high spatial resolution of activity as a result of multiple simultaneous recordings from multiple hippocampal structures. The results of our spatio-temporal analysis suggest that there may be an important difference in the way pyramidal cells are interconnected in CA3c, and that this region may be involved in the generation of high frequency oscillations in hippocampus, which may contribute to the epileptogenic properties area CA3 in hippocampus. The difference in connectivity likely reflects quantitative difference in either strength or frequency of excitatory connections.

This quantitative difference in synaptic connectivity is further supported by our data that oscillations were less pronounced in CA1 after CA3 was physically removed from the slice by microknife cut. As illustrated, HFOs occurred as part of the CA1 events, but the amplitude was less and higher frequency features were evident as a result of activity losses in a manner consistent with the mechanism proposed by Foffani et al. (33).

With a relatively high density recording array, the laminar profiles of HFO are available in each structure together with accurate timing data for studies of activity spread. The variance in proposed mechanisms and locations of origin suggest that multiple forms of HFOs may exist in the hippocampus, but our finding of very similar properties for HFOs facilitated by disinhibition or by glutamate receptor activation suggests that there may be regional differences that emerge when the primary initiation point is removed.

Our data include area CA2 in nearly all recordings (see Figures 1, 2, 7 as examples). Whereas, Oliva et al. (35) showed that CA2 appeared to be the origin for synchronous activity, in our recordings, CA2 did not lead CA3c in the oscillations no matter what the stimulus location was nor if the HFOs were part of spontaneous events.

One possible explanation for the localization of oscillations in area CA3 is the likelihood that mossy fiber axons have the highest density in this part of the slice. In addition, axonal gap junctions have been demonstrated in mossy fibers (26) and may contribute to pyramidal cell synchronization. Proximal CA3b and the CA3c subregions send their axons predominantly to the CA1 region. A fraction of collaterals also project to the dentate gyrus (36, 37). Our spatial account of the oscillations can be explained, therefore, on the basis of hippocampal connectivity.

Interestingly, the study by Foffani et al., which demonstrated the emergence of very high frequency oscillations from HFOs or ripple activity (33) also points to CA3c as a spatial focal point. As HFOs are the required substrate for very high frequency oscillations, it is to be expected that their spatial localization overlaps. Further, this points to a linkage between normal HFOs and what may be considered pathological very high frequency oscillations.

### Synaptic Control of High Frequency Oscillations

Our study indicates that either GABA_A_ inhibition or activation of kainate receptors is sufficient for the emergence of robust HFOs. Our data indicate that oscillations require AMPA receptors, but not NMDA receptors, the latter of which has previously been shown (17). Our findings are further supported by evidence that HFOs are dependent on both inhibitory and excitatory control (20), and they can thus be driven by loss of one or gain of the other.

In optogenetically induced HFOs, loss of excitation of increases in inhibition aborted the oscillations (38). The increased frequency of the oscillations while inhibition is still present in the slice illustrates a paradoxical effect of inhibition of increasing the circuit's frequency. The difference in duration of the events under disinhibition and excitation suggests an intrinsic oscillatory circuit that is modulated more by inhibition than by limitation of excitation. This may be related to the observation that HFOs occurred spontaneously in the presence of kainic acid but not bicuculline.

HFO activity was triggered from multiple sites within hippocampus (different subregions and different layers within subregions) and all stimulus sites led to the same finding that oscillations appeared to originate in area CA3c (see Figure 3). Direct and antidromic activation of neurons certainly occurred with our single pulse stimulation. The best evidence for this is the single population spike that remains after CNQX exposure (Figure 10). The long latency for population events containing high frequency oscillations (Figure 1) when stimuli were applied to CA1 is another indicator that cell-to-cell connectivity (synaptic or otherwise) is necessary for the generation of events containing HFOs.

### Gap Junctions Affect Neuronal Activity but Not Neuronal Synchrony

Our work shows that while gap junctions may impact the frequency of firing of neurons in a population, the synchronization of that activity does not appear to require gap junctions. Specific gap junction blockade cannot be done with precision with any available drug, and therefore, while a number of gap junction “blockers” can stop HFOs (17), this may be due to other effects of the various gap junction blockers. Gap junction activation in the presence of glutamate blockade did not aid in HFO formation, but did increase the overall amount of neuronal activity. Gap junction blockade clearly reduced the amount of neuronal activity (Figure 10C). These results are consistent with the view that both a means to generate activity and a means to synchronize such activity are necessary for population oscillations. Our findings clearly illustrate how gap junction activity can contribute to the presence of neuronal activity that might become synchronous, but gap junctions do not appear to be the synchronization mechanism. Glutamate receptors appear to be the critical synchronization mechanism. We speculate that if gap junctions do exist in mossy fibers at mixed chemical and electric synapses and the mossy fiber density is greatest in CA3 (26), gap junctions in area CA3c may thus account for our observation that HFOs originate and have such large amplitudes in area CA3c.

### Clinical Significance

HFOs are known to occur frequently in mesial temporal lobe epilepsy (39). These areas are additionally thought to be an indicator of the seizure onset zone independent of interictal spikes (40). Further, seizure outcomes have been found to be better with removal of a larger extent of tissue with HFOs (41). However, the scale at which HFOs are detected cannot easily resolve particular hippocampal substructures. Our work contributes to the idea that HFOs arising within the mesial temporal lobe reflect epileptogenicity in that we show a specific circuit that is pre-disposed to HFO generation in the setting of abnormal excitation or disinhibition. We propose that this intrinsic circuit may play a role in the generation of HFOs and epileptogenicity in mesial temporal lobe epilepsy.

### Limitations

One limitation of this work is that while HFOs exist in normal and pathologic contexts, this study uses a brain slice model to study them. Additionally, the effects of synaptic disinhibition or excitation on generation of HFOs may not necessarily reflect synchrony in the generation of seizures. However, this study does nonetheless describe an intrinsic circuit that may be important in seizure generation.

## Data Availability Statement

The datasets generated for this study are available on request to the corresponding author.

## Ethics Statement

The animal study was reviewed and approved by Animal Care and Use Committee, SUNY Downstate Medical Center.

## Author Contributions

RO designed the study and performed the experiments. MS and IN performed the data analysis. RO and IN prepared the figures. IN wrote the manuscript, which was revised by RO, MS, and IN.

## Conflict of Interest

The authors declare that the research was conducted in the absence of any commercial or financial relationships that could be construed as a potential conflict of interest.
